# Cardiac Fibroelastoma: A Rare Cause of Stroke in Young Adults

**DOI:** 10.1155/2013/250808

**Published:** 2013-05-13

**Authors:** Ahmad Mirdamadi, Mohsen MirmohammadSadeghi, Mahfar Arasteh, Mojgan Gharipour

**Affiliations:** ^1^Medical School, Islamic Azad University, Najafabad Branch, Isfahan, Iran; ^2^Hypertension Research Center, Isfahan Cardiovascular Research Institute, Isfahan University of Medical Sciences, Isfahan, Iran; ^3^Isfahan University of Medical Sciences, Isfahan, Iran; ^4^Isfahan Cardiovascular Research Center, Isfahan Cardiovascular Research Institute (WHO Collaborating Center), Isfahan University of Medical Sciences, Isfahan, Iran

## Abstract

A 26-year-old man presenting with a transient episode of dysarthria and dizziness, 3 weeks prior to admission, was referred to our center to be evaluated for transient ischemic attack (TIA). The patient had been previously admitted to a different hospital and echocardiography was reported normal at that center, but upon presenting to our institution strand-like masses in the left ventricle (LV) were detected. Transesophageal echocardiography (TEE) revealed two distinct mobile LV masses suggesting a diagnosis of papillary fibroelastoma. CT angiography and histopathological studies confirmed this diagnosis.

## 1. Case Report

**Figure 1:**
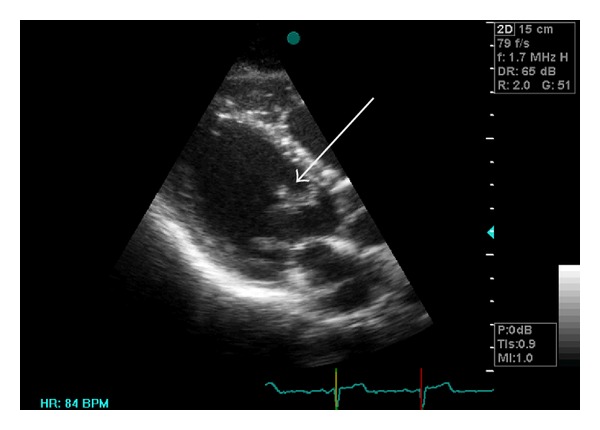
Evidence of strand-like LV masses, as shown by TTE.

**Figure 2:**
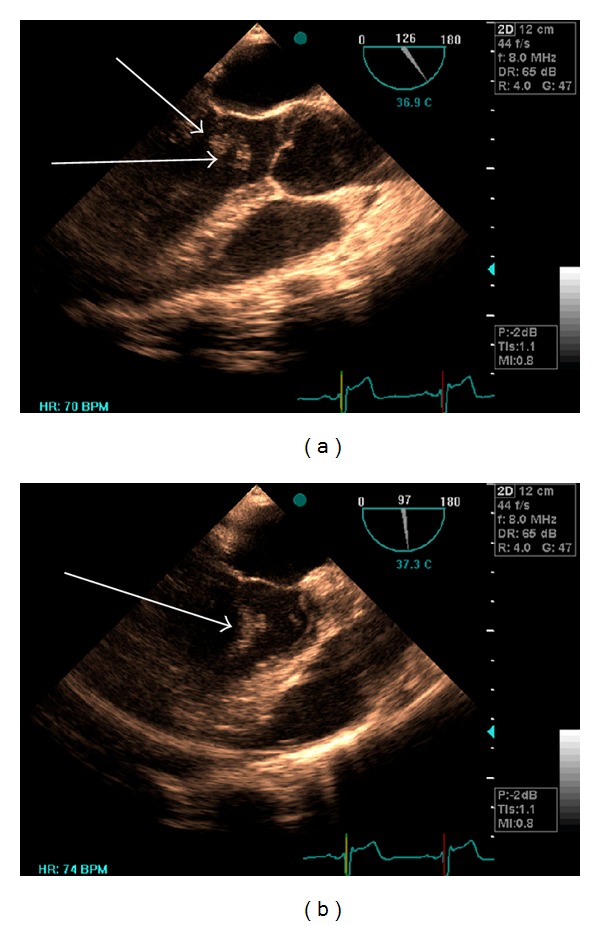
Transesophageal echocardiographic image showing mobile LV masses attached to the mitral valve and the posterior wall extending to LV outflow tract.

**Figure 3:**
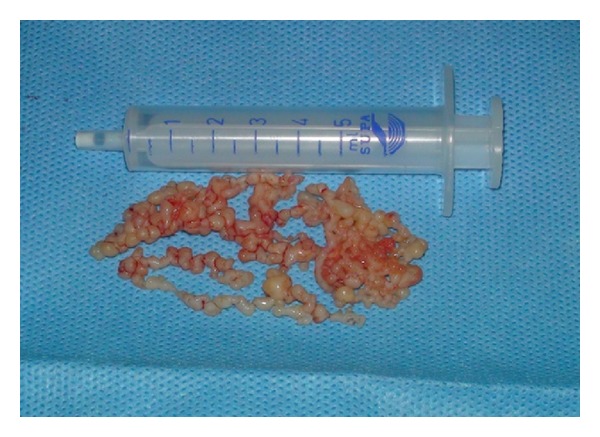
Gross specimen of papillary fibroelastoma demonstrating two large beading tumors.

A 26-year-old man presenting with an episode of dysarthria and dizziness 3 weeks prior to admission was referred to our center to be evaluated for transient ischemic attack (TIA). His past medical history showed no evidence of previous illness. On admission, physical examination showed that he was in sinus rhythm, had a pulse rate of 75 bpm, and a blood pressure of 100/60 mm Hg. On auscultation, he had no murmur or carotid bruit. Laboratory tests were normal. He had a normal electrocardiogram and chest X-ray. Initial cardiac evaluation was also normal. The patient had been previously admitted to a different hospital which had performed transthoracic echocardiogram and reported it as normal. The patient was referred for transesophageal echocardiography (TEE) to rule out of patent foramen ovale (PFO). We performed another transthoracic echocardiogram (TTE), but mobile strand-like masses in left ventricular (LV) were detected (Figure 1). TEE also revealed two distinct highly mobile filamentous LV masses, suggesting papillary fibroelastoma (Figures 2(a) and 2(b)). One mass measuring 5 × 0.6 cm originated from the posterior wall, near the apex, and another mass measuring 3 × 0.6 cm originated from the mitral valve (MV) annulus just beside the posterior MV leaflet. Both of the masses extended toward LV outflow tract. Both aortic and mitral valve were spare, but chordae tendina was suspected to be involved. CT angiography confirmed the diagnosis. The patient was given anticoagulant therapy and referred for surgery. At operation, two large beading tumors were found in the LV (Figure 3). One of the tumors was attached to posterior leaflet of the MV and the other one was attached to the posterior papillary muscle adjacent to the chordae tendina. The tumors were successfully excised via mitral valve approach. Chordae reconstruction and MV annuloplasty were performed for the patient. The postoperative recovery was uneventful. Follow-up TEE revealed no residual tumor.

## 2. Discussion

Fibroelastomas are small, avascular, and usually solitude lesions (in 91% of cases) [1]. This tumor is a very rare tumor with an incidence of 0.001–0.3% at autopsy [2, 3], and most fibroelastomas are often diagnosed incidentally [4–7]. In symptomatic patients, the clinical presentation depends on the location of tumor [8]. The most common clinical presentation is stroke or transient ischemic attack (TIA) caused by cerebral emboli, originating from a thrombus on the tumor, or by a piece of the tumor itself. Unfortunately, the cerebral ischemia could also result in completed strokes [8]. They have multiple papillary fronds resembling sea anemones. Since the symptoms may mimic other conditions and the fibroelastoma has a similar echocardiographic appearance to thrombus and other various types of cardiac mass, the tumor may be not considered as a diagnosis or it may erroneously be regarded as a thrombus, vegetation, or degenerative valve tissue [9, 10].

In our case, the first transthoracic echocardiogram was not able to correctly diagnose the fibroelastoma and only after meticulous image acquisition, in a second echocardiogram, a correct diagnosis was made. The diagnosis of the fibroelastoma was confirmed by CT angiography and histology after surgery. In cases of cardiac tumor, TEE has an important role in making the diagnosis [11, 12]. Echocardiographic diagnosis can be very difficult, particularly when there is no definitive characteristic to distinguish fibroelastomas from other masses or when the acoustic window is not adequate. When the tumor is larger than 0.2 cm, the sensitivity and specificity of TTE are 88.9% and 87.8%, respectively [10]. Additionally, multiple fibroelastomas are only detected in 8.6% of cases by TEE [10]. Therefore, there should be high clinical suspicion to consider the possibility of a cardiac tumor and careful attention should be paid in order to find these masses in echocardiography. TEE should be performed to make the appropriate diagnosis.
